# Effect of direct oral anticoagulants on bleeding during and after cataract surgery

**DOI:** 10.1007/s10792-024-02944-x

**Published:** 2024-02-20

**Authors:** Anat Maytal, Hadar Naidorf Rosenblatt, Reut Rotem, Fani Segev

**Affiliations:** 1 Department of Ophthalmology, Meir Medical Center, Meir Hospital, 59 Tchernichovsky St, 4428164 Kfar Saba, Israel; 2 Sackler Faculty of Medicine, Tel Aviv University, Tel Aviv, Israel; 3 Department of Ophthalmology, Kaplan Medical Center, Rehovot, Israel; 4 Department of Obstetrics and Gynecology, Shaare Zedek Medical Center, Jerusalem, Israel; 5 Department of Ophthalmology, Assuta-Samson Medical Center, Ashdod, Israel; 6 Joyce and Irwing Goldman Medical School, Ben-Gurion University, Beer-Sheva, Israel

**Keywords:** Direct oral anticoagulants, NOACS, Apixaban, Intraoperative bleeding, Hyphema, Thromboembolic event

## Abstract

**Purpose:**

To assess the risk for intraoperative and postoperative ocular bleeding associated with direct oral anticoagulant treatment in patients undergoing phacoemulsification surgery.

**Methods:**

Consecutive patients had phacoemulsification and intraocular lens implantation while taking uninterrupted direct oral anticoagulants (dabigatran, rivaroxaban, or apixaban). Gender and age-matched patients without antithrombotic therapy were used as the control group. Patients were examined one week postoperatively. Intraoperative and postoperative hemorrhagic and non-hemorrhagic complications were assessed.

**Results:**

Forty patients (56 eyes) on direct oral anticoagulants and 120 patients (172 eyes) without anticoagulation, at a mean age of 77 years, had phacoemulsification. There was no significant difference between the groups in the rate of intraoperative and postoperative bleeding. One eye (1.8%) in the treatment group and 3 eyes (1.7%) in the control group had hyphema (*p* = 0.72). No patient had thromboembolic event during or after surgery.

**Conclusions:**

Cataract surgery was safely performed while continuing direct oral anticoagulation.

## Introduction

Direct oral anticoagulants (DOACs) were developed as alternatives to warfarin. The most common available DOACs are the direct factor IIa inhibitor dabigatran (Pradaxa), and the direct factor Xa inhibitors rivaroxaban (Xarelto) and apixaban (Eliquis) [1, 2]. As the population ages, antithrombotic drugs are widely used, and DOACs are licensed for thromboprophylaxis after orthopedic surgeries, treatment and secondary prophylaxis of venous thromboembolism, and for prevention of stroke in non-valvular atrial fibrillation [2–4].

Warfarin inhibits the vitamin K pathway, and therefore affects numerous clotting factors [1]. DOACs provide more predictable anticoagulation with rapid onset of action, shorter half-lives and fixed dosing, and are less prone to interact with other drugs [1, 2, 5]. Therefore, they provide safer and at least as effective anticoagulation without the need for frequent coagulation monitoring [6–8].

Phacoemulsification with intraocular lens (IOL) implantation is considered an avascular procedure with less than 3% risk of bleeding, and less than 0.1% chance of sight-threatening bleeding [9–12]. Numerous studies have investigated the risk of hemorrhagic complications in patients undergoing cataract extraction while taking uninterrupted warfarin [10, 11, 13–15]. Continuing anticoagulant therapy has consistently shown no increased risk for sight-threatening anesthetic or operative complications [10, 11]. Given the thrombotic risk in these anticoagulated patients, some evidence-based guidelines recommend continuing warfarin in patients undergoing cataract surgery [9, 16].

Although DOACs are widely used, data regarding using them perioperatively are lacking. This study describes our experience with uninterrupted DOACs during cataract surgery, and is one of the few to examine this approach adequately.

## Methods

The study protocol was approved by the Meir Medical Center Institutional Review Board (Helsinki Committee). Informed consent was not required due to the retrospective nature of the data.

A retrospective review of electronic medical records of consecutive patients older than 50 years treated with DOACs (dabigatran, rivaroxaban and apixaban), and undergoing phacoemulsification and IOL implantation during the study period of January 2017–July 2019 at Meir Medical Center, was conducted. We then examined gender and age-matched controls without anticoagulation, in a ratio of 3 for each anticoagulated patient. Patients undergoing general or retrobulbar anesthesia, or using other type of anticoagulants or antiaggregants during the time of the surgery (including combined therapy with DOACs), were excluded. Other exclusion criteria were traumatic cataract, posterior synechiae, rubeosis iridis, subluxated or luxated crystalline lens, and cataract extraction combined with another type of ocular surgery (vitrectomy, filtration surgery, etc.).

Patient demographics, systemic and ocular co-morbidities, CHA_2_DS_2_ VASc score, renal function, type of DOAC, operative risk factors (cataract density, pupil size, use of pupil expansion devices, anterior chamber depth, axial length, and systemic alpha blocker treatment), type of anesthesia, preoperative and postoperative distance visual acuities, intraoperative and postoperative complications and systemic complications within 7–10 days after surgery, were recorded.

In our department, the common practice is to continue all blood thinners during cataract surgery. Hence, at the preoperative assessment clinic, patients on DOAC medications were advised to continue their treatment.

All surgeries were done under local anesthesia using topical 0.4% oxybuprocaine (Localin; Fischer pharmaceuticals, Bnei Brak, Israel) combined with intracameral anesthesia with up to 1.5 ml of 1% lidocaine (Esracain; Rafa Laboratories, Jerusalem, Israel). Clear corneal incisions were typically 2.2–2.4 mm wide. Phacoemulsification was performed using the Infiniti Vision System (Alcon Laboratories, TX, USA). Preventive intracameral cefuroxime was injected at close of surgery in all cases without contraindications.

Postoperative treatment consisted of topical steroids and antibiotics. In addition, topical nonsteroidal anti-inflammatories were given to patients at risk of macular edema, such as patients with diabetes, after retinal vein occlusion or a significant surgical complication. Patients were seen 1 day and 7–10 days postoperatively, unless more frequent or prolonged follow-up was required.

### Data analysis

Intraoperative and postoperative bleeding were considered as the primary dependent variables. In order to identify differences between patients treated with DOACs and patients not treated with DOACs during cataract surgery, including differences in the rate of bleeding, *T* test was used for continuous variables, χ^2^ test (or Fisher’s exact test) was used for categorical variables, and Mann–Whitney test was used for ordinal variables (or whenever normality assumption was not satisfied). For analysis, visual acuities were converted to logMAR values. Off-chart acuities were converted to logMAR as described by Schulze-Bonsel, et al. [17]. Data were analyzed using SPSS software (version 23.0; SPSS Inc., Chicago, IL, USA). *P* values of ≤ 0.05 were considered statistically significant.

## Results

The study group included 40 patients who had surgery while taking uninterrupted DOAC medications. Table 1 elaborates the type of DOAC that was administrated and indications for treatment. Fifty-seven percent (23/40) of the patients were under apixaban treatment and 80% (32/40) had atrial fibrillation. The control group included 120 age and gender-matched patients without antithrombotic therapy. Sixty percent of both groups (24/40 in the study group and 72/120 in the control group) were male, and the mean age was 77.3 years in the study group and 77.2 years in the control group.

**Table 1:** Direct oral anticoagulant (DOAC) therapy

Variable	Patients (n = 40) n (%)
DOAC agent	
Dabigatran	6 (15%)
Rivaroxaban	11 (27.5%)
Apixaban	23 (57.5%)
Indication for DOAC treatment	
Atrial fibrillation	32 (80%)
Venous thromboembolism secondary prevention	2 (5%)
Unknown or other indication	6 (15%)

Table 2 shows the systemic clinical characteristics of the patients. Hypertension, ischemic heart disease, heart failure, and cerebrovascular disease rates were all significantly higher in the study group, as were CHA_2_DS_2_ VASc scores and creatinine levels.

**Table 2:** Patient characteristics

Characteristic	Study group (n = 40)	Control group (n = 120)	*P* value
Eyes, n	56	172	–
Systemic co-morbidities			
Diabetes mellitus, *n* (%)	12 (30%)	44 (36.7%)	0.44
Hypertension, *n* (%)	31 (77.5%)	66 (55.0%)	0.02
Hyperlipidemia, *n* (%)	24 (60.0%)	57 (47.9%)	0.30
Ischemic heart disease, *n* (%)	18 (45.0%)	8 (6.7%)	< 0.01
Heart failure, *n* (%)	6 (15.0%)	4 (3.3%)	< 0.01
Cerebrovascular disease, *n* (%)	12 (30%)	1 (0.8%)	< 0.01
CHA_2_DS_2_ VASc score, median (IQR)	4 (4–5.75)	3 (2–4)	< 0.01
Creatinine (mg/dL), mean ± SD	1.07 ± 0.37	0.93 ± 0.33	0.04

*IQR* interquartile ratio

Fifty-six eyes in the study group and 172 eyes in the control group underwent cataract surgery during the study period. Both groups were similar in terms of preoperative visual acuities and ocular co-morbidities (Table 3). Median preoperative visual acuity was 6/15 in both groups. Pseudoexfoliation syndrome and glaucoma were the most prevalent ocular diseases. There were no statistically significant differences in most surgical risk factors, including cataract density, pupil size, and axial length. However, systemic alpha blocker treatment rate was significantly higher in the study group—35.7% (20/56) versus 15.1% (26/172), *p* = 0.01.

**Table 3:** Ocular characteristics

Characteristic	Study group (n = 56 eyes)	Control group (n = 172 eyes)	*P* value
Preoperative logMAR VA, median (IQR)	0.40 (0.30–0.52)	0.40 (0.30–0.70)	0.52
Ocular co-morbidities			
Age-related macular degeneration, n (%)	1 (1.8%)	15 (8.7%)	0.80
Diabetic retinopathy, n (%)	3 (5.4%)	3 (1.7%)	0.14
Glaucoma, n (%)	8 (14.3%)	16 (9.3%)	0.29
Pseudoexfoliation, n (%)	12 (21.4%)	32 (18.6%)	0.64
Corneal opacity, n (%)	3 (5.4%)	6 (3.5%)	0.53
Amblyopia, n (%)	1 (1.8%)	4 (2.3%)	0.81
Alpha blocker treatment, n (%)	20 (35.7%)	26 (15.1%)	< 0.01
Nuclear sclerosis grade, median (IQR)	2 (2–3)	2 (2–3)	0.46
Pupil diameter (mm), mean ± SD	6.8 ± 0.95	7.1 ± 1.05	0.15
Use of pupil expansion device, n (%)	0 (0.0%)	2 (1.2%)	0.41
Axial length (mm), mean ± SD	23.31 ± 0.96	23.46 ± 1.20	0.40
Anterior chamber depth (mm), mean ± SD	2.93 ± 0.45	3.1 ± 0.42	0.05
IOL implantation position			
Capsular bag, n (%)	54 (96.4%)	166 (96.4%)	0.91
Ciliary sulcus, n (%)	1 (1.8%)	4 (2.4%)	
Anterior chamber, n (%)	1 (1.8%)	2 (1.2%)	

*VA* visual acuity, *IQR* interquartile ratio

The rate of intraoperative non-hemorrhagic complications was similar in both groups (Table 4). The treatment group showed a nonsignificant trend to intraoperative floppy iris syndrome (IFIS), as compared to the control group. Intraoperative hyphema was noted in 3 cases: 1 eye of the study group and 2 eyes of the control group. All 3 hyphemas were secondary to iatrogenic iris trauma. There were no other intraoperative hemorrhagic complications, including vitreous, choroidal, suprachoroidal or retrobulbar. Postoperative non-hemorrhagic complications were also similar between the groups, with corneal edema being the most common complication (reported in 11 eyes in the study group and 38 eyes in control group). Postoperative hemorrhages included eyelid hematoma in one eye, subconjunctival hemorrhage in 3 eyes, hyphema in 4 eyes (all but one were noted intraoperatively), vitreous hemorrhage in one eye, and retinal hemorrhage in one eye, with no significant difference in the rate of bleeding between groups. There were 2 eyes in the study group and 5 in the control group with any hemorrhagic complication (3.6% vs. 2.9%. respectively; *p* = 0.57). There was no difference in postoperative visual acuities and no reported systemic complications during or after surgery.

**Table 4:** Intra- and postoperative complications and postoperative visual acuity

Complications	Study group (n = 56 eyes)	Control group (n = 172 eyes)	*P* value
Intraoperative			
Capsulorhexis tear, n (%)	0 (0.0%)	2 (1.2%)	0.56
Posterior capsule rapture, n (%)	2 (3.6%)	8 (4.7%)	0.53
Dropped nucleus, n (%)	0 (0.0%)	3 (1.7%)	0.42
Zonulolysis, n (%)	0 (0.0%)	2 (1.2%)	0.42
Iris trauma, n (%)	1 (1.8%)	2 (1.2%)	0.72
IFIS, n (%)	8 (14.3%)	11 (6.4%)	0.06
Hyphema, n (%)	1 (1.8%)	2 (1.2%)	0.57
Systemic, n (%)	0 (0.0%)	0 (0.0%)	–
Postoperative			
Corneal edema, n (%)	11 (19.6%)	38 (22.1%)	0.54
Toxic anterior segment syndrome, n (%)	0 (0.0%)	1 (0.6%)	0.55
Residual lens material, n (%)	0 (0.0%)	2 (1.2%)	0.55
Eyelid hemorrhage, n (%)	1 (1.8%)	0 (0.0%)	0.26
Subconjunctival hemorrhage, n (%)	1 (1.8%)	2 (1.2%)	0.59
Hyphema, n (%)	1 (1.8%)	3 (1.7%)	0.72
Vitreous hemorrhage, n (%)	0 (0.0%)	1 (0.6%)	0.74
Retinal hemorrhage, n (%)	0 (0.0%)	1 (0.6%)	0.55
Systemic, n (%)	0 (0.0%)	0 (0.0%)	–
Total hemorrhagic complications, n (%)	2 (3.6%)	5 (2.9%)	0.57
Postoperative logMAR VA, median (IQR)	0.30 (0.15–0.40)	0.22 (0.15–0.40)	0.83

*IFIS* intraoperative floppy iris syndrome, *VA* visual acuity, *IQR* interquartile ratio

## Discussion

This study showed that a strategy of uninterrupted DOACs is feasible and safe during cataract surgery.

Compared to warfarin, DOACs are considered safer and cause less spontaneous bleeding, including intraocular bleeding [6–8, 18, 19]. The two concerns with the DOACs are that their anticoagulant effect is difficult to reliably measure with standard coagulation tests, and their elimination is affected by renal function [2, 3, 20].

There is lack of experience in managing patients treated with DOACs who need to undergo elective or emergency surgery [3]. In the RE-LY trial [20], 4591 patients with atrial fibrillation treated with dabigatran or warfarin, required treatment interruption for a total of 7637 surgeries or procedures, including 427 patients who underwent cataract extraction during the study period. Dabigatran and warfarin were associated with overall similar rates of periprocedural bleeding and thrombotic complications. Subgroup analysis according to the specific type of the procedure was not performed, and there was no comparison to continuous anticoagulation.

Information addressing the specific risk for hemorrhagic complications during and after cataract surgery related to DOACs is very limited. Patel et al. [21] reviewed 252 cases using a policy of withholding one dose of DOAC before cataract surgery and then renewing it 6 h after the procedure. No thromboembolic events occurred. Cheung et al. [22] described 66 eyes of 53 patients, of which 42 continued and 24 withheld DOAC therapy before surgery. There was no statistically significant difference between the two groups in intraoperative, postoperative and systemic complications, with only two cases of bruising and subconjunctival hemorrhage in the withheld group, and specifically in patients receiving retrobulbar anesthesia.

In our department, the accepted practice is to continue all antithrombotic agents during simple phacoemulsification surgery. The main purpose of this study was to compare the rates of intraoperative and postoperative hemorrhage between patients treated with DOACs and patients without anticoagulation. We found no additional risk for hemorrhagic complications, as compared to the standard risk of cataract surgery itself.

Study and control groups were different in terms of systemic co-morbidities, with a higher rate of hypertension, ischemic heart disease, heart failure, and cerebrovascular disease, and higher median of CHA_2_DS_2_ VASc score, in the study group. This is to be expected, since anticoagulation is indicated for stroke prevention only in high-risk patients with atrial fibrillation [23]. Renal function was found to be significantly worse in the study group. This could be explained by the aforementioned higher proportion of patients with hypertension and cardiovascular disease, both related to chronic kidney disease [24]. Benign prostatic hyperplasia (BPH) is also associated with hypertension and metabolic syndrome [25]. This can explain why alpha-1-adrenergic antagonist therapy, often prescribed to treat lower urinary tract symptoms secondary to BPH, was more common in the study group [26]. Even though these anticoagulated patients had more systemic co-morbidities, they did not have a higher rate of perioperative complications.

The most common non-hemorrhagic intraoperative complication was IFIS, noted in 14.3% of DOAC-treated eyes and in 6.4% of control eyes (*p* = 0.06). This close-to-significant difference was probably due to the higher proportion of alpha blocker treatment, a major risk factor for IFIS, in the study group, as mentioned [26].

A total of 7 eyes had any hemorrhagic complication: 2 in the study group and 5 in the control group. Of these, 4 eyes (1 DOAC group and 3 controls), were reported with hyphema; 3 cases were noted intraoperatively and a fourth case about a week later. The overall rate of hyphema was high compared to previous reports [10, 11]. All 4 patients with hyphemas underwent complicated procedures, with posterior capsule rupture and iatrogenic injury to the iris and in 2 cases, dropped nucleus into the vitreous cavity. One case had an additional vitreous hemorrhage noted postoperatively. Final visual acuity for 3 of the cases was good. One required lamellar endothelial keratoplasty 5 months later for persistent corneal edema.

Several studies show a higher incidence of subconjunctival hemorrhage in patients receiving uninterrupted warfarin, but these were self-limiting without compromise to visual acuity [11, 15]. One of the strategies to reduce subconjunctival bleeding, is to use topical brimonidine preoperatively. Desco et al. [27] had 80 patients receiving brimonidine shortly before phacoemulsification, with much lower rate of subconjunctival hemorrhages compared to untreated patients. There was no difference with regard to the presence of hemorrhages in patients with anticoagulant or antiplatelet treatment; however, they used peribulbar anesthesia in all cases and discontinued all blood thinners before surgery. Ucar et al. [28] used preoperative brimonidine to decrease intraoperative and postoperative hemorrhage related to trabeculectomy, a much bloodier procedure. They used a vision analysis software to demonstrate a quantitative significant reduction in conjunctival redness after surgery. In our study, we had only 3 cases of subconjunctival hemorrhages, despite not using any special technique or withholding anticoagulants, one in the treatment and 2 in the control group.

There were no other sight-threatening hemorrhagic complications and no systemic events, including cerebrovascular event, acute coronary syndrome or venous thromboembolism, in either group.

European, American and international guidelines regarding continuing or stopping DOACs prior to surgeries with low risk of bleeding varies, due to relative lack of experience and large-scale data using these drugs perioperatively. The American College of Cardiology published a clinical expert consensus decision pathway [29] for periprocedural management of anticoagulation in patients with non-valvular atrial fibrillation, and suggested that procedures routinely performed with a very low risk of bleeding, are best performed with no or limited interruption of DOACs. The European Heart Rhythm Association [30] similarly suggested, that in procedures that carry ‘no clinically important bleeding risk’, DOACs can be taken without interruption, scheduling the procedure at the predicted nadir of the drug level, or skip only one dose. Guidelines from the ophthalmology community are lacking, although some hospitals have adopted a policy of allowing patients to continue DOACs through cataract surgery [1, 21].

This study is the first to address properly the perioperative risk of ocular bleeding during and after cataract surgery, related to DOACs. The study is limited due to its retrospective nature and relatively small sample size. We did not compare the rate of complications between continuing and withholding DOACs.

In conclusion, the present study demonstrates that in patients undergoing phacoemulsification surgery, there is no significant difference in hemorrhagic complications between those continuing DOACs perioperatively as compared to patients without anticoagulation therapy. Uninterrupted use of DOACs appears safe, and should be weighed against the thrombotic risk of withholding treatment in these patients. Further large-scale studies are needed to establish evidence-based guidelines.
